# The Function of Lipin in the Wing Development of *Drosophila melanogaster*

**DOI:** 10.3390/ijms20133288

**Published:** 2019-07-04

**Authors:** Tran Duy Binh, Tuan L. A. Pham, Taisei Nishihara, Tran Thanh Men, Kaeko Kamei

**Affiliations:** 1 Department of Functional Chemistry, Kyoto Institute of Technology, Kyoto 606-8585, Japan; 2 Department of Biology, Can Tho University, Cantho City 900000, Vietnam

**Keywords:** *Drosophila*, lipin, DNA damage, cell cycle, wing development

## Abstract

Lipin is evolutionarily conserved from yeast to mammals. Although its roles in lipid metabolism in adipocyte tissue, skeletal muscle, and the liver, and as a transcriptional co-activator are known, its functions during development are still under investigation. In this study, we analyzed the role of *Drosophila* lipin (dLipin) in development. Specifically, we showed that the tissue-selective knockdown of *dLipin* in the wing pouch led to an atrophied wing. Elevated DNA damage was observed in the wing imaginal disc of *dLipin*-knockdown flies. dLipin dysfunction induced accumulation of cells in S phase and significantly reduced the number of mitotic cells, indicating DNA damage-induced activation of the G2/M checkpoint. Reduced expression of cyclin B, which is critical for the G2 to M transition, was observed in the margin of the wing imaginal disc of *dLipin*-knockdown flies. The knockdown of *dLipin* led to increased apoptotic cell death in the wing imaginal disc. Thus, our results suggest that dLipin is involved in DNA replication during normal cell cycle progression in wing development of *Drosophila melanogaster*.

## 1. Introduction

The phospholipid components of biological membranes are pivotal for cellular processes including growth, differentiation, and transport, as phospholipids participate in important signaling cascades [1,2,3]. Phospholipid synthesis involves phosphatidic acid (PA) and diacylglycerol (DAG), both of which have critical roles in signaling cascades, energy storage, and lipid anabolism pathways [4]. Carman et al. (2017) showed that PA is degraded by conversion into DAG, the direct precursor for producing phosphatidylcholine and phosphatidylethanolamine [5]. Moreover, PA can be converted to cytidine diphosphate diacylglycerol (CDP-DAG), the precursor for the production of PA, phosphatidylglycerol, cardiolipin, and phosphatidylinositol [6]. They inhibit the reactions of DNA polymerases, which are essential for DNA replication [7].

In the cytoplasm, lipins function as a type of phosphatidate phosphatases (PAP1 enzymes) that catalyze Mg^2+^-dependent dephosphorylation of PA to form DAG at the endoplasmic reticulum (ER) membrane. Thus, lipins have an essential role in coordinating the balance between PA and DAG [8] and are involved in the formation of triacylglycerol (TAG) [9], which plays a central role in cellular lipid storage [10]. In the nucleus, lipin works as a transcriptional co-activator in a complex with peroxisome proliferator-activated receptor γ coactivator-1α (PGC-1α) and peroxisome proliferator-activated receptor α (PPARα) [11,12], which are master regulators of genes related to mitochondrial biogenesis and fatty acid oxidation [11,12]. The function of lipins is evolutionarily conserved from eukaryotes to mammals [13]. In humans, the lipin protein family consists of three members: lipin 1, 2, and 3 [14,15], which are localized within different tissues. Lipin 1, the best characterized among the three, resides in the fat tissues and cardiac and skeletal muscles, whereas lipin 2 and lipin 3 are detected in the liver [16,17,18] and intestine [17], respectively. A previous study demonstrated that lipins 1 and 2 in mammalian cells are inhibited by phosphorylation during mitosis, causing a reduction in the cellular PAP1 activity during cell division [13]. This study suggested the possibility that a decrease in PAP1 activity could contribute to the inhibition of phospholipid accumulation prior to cell division. In yeast, lipin can negatively control the synthesis of phosphatidylcholine and other phospholipids by suppressing key phospholipid biosynthesis pathway genes [11]. The subsequent abnormalities of phospholipid synthesis may indirectly affect DNA replication [19]. Moreover, loss of lipin induces the overgrowth of intracellular membranes, affects the envelopes of nuclei and peripheral ER, and leads to defective chromosome segregation [11,20]. Genetic knockdown of *lipin 1* in mice induces lipodystrophy and insulin resistance and alters hepatic metabolism [21], whereas transgenic mice overexpressing lipin 1 show an obese phenotype [22]. In *Drosophila*, decreased expression of *Drosophila* lipin (dLipin) was found to affect the normal development of the fat body, which is the major tissue for TAG storage in invertebrates [23], and resulted in down-regulation of the insulin-receptor-controlled PI3K-Akt pathway and increased hemolymph sugar levels [24]. Schmitt et al. indicated that insulin and target of rapamycin complex 1 (TORC-1) pathways independently regulate nuclear translocation of dLipin [24]. In mammals, blocking TORC1 dephosphorylates lipin 1, leading to its translocation from the cytoplasm into the nucleus, where it affects nuclear protein levels, but not mRNA levels, of the transcription factor sterol regulatory element-binding protein 1 (SREBP1), which is a main regulator of genes that are related to the biosynthesis of fatty acid, cholesterol, TAG, and phospholipid [25].

The cell cycle consists of a series of events that lead to cell division and the duplication of cellular DNA, which is then precisely separated into daughter cells. There are two main regulators of cell cycle progression, cyclins and cyclin-dependent kinases (CDKs) [26]. Cyclins are divided into four classes. G1/S cyclins, S cyclins, and M cyclins are directly related to the control of cell cycle events, whereas G1 cyclins control the entry into the cell cycle in response to extracellular growth factors and mitosis [27]. CDKs contain a serine/threonine-specific catalytic core and associates with cyclins to regulate kinase activity and substrate specificity [28], promoting S phase progression, checkpoint, and mitosis [26,27,29,30]. For example, CDK2 is important for S phase progression whereas CDK1 is essential for the G2 checkpoint and mitosis [31,32,33]. The cell cycle contains several specific checkpoints to monitor and control its progression and to allow verification of phase processes and repair of DNA damage [34,35,36]. There are three specific checkpoints for damaged or incompletely replicated DNA: G1/S, G2/M, and intra-S checkpoints [27]. The current study aimed to reveal the role of lipins in development using *Drosophila melanogaster*. Specifically, we investigated the role of dLipin in cell cycle progression during wing formation in *D. melanogaster*.

## 2. Results

### 2.1. Localization of dLipin in Wing Imaginal Disc

**Figure 1:**
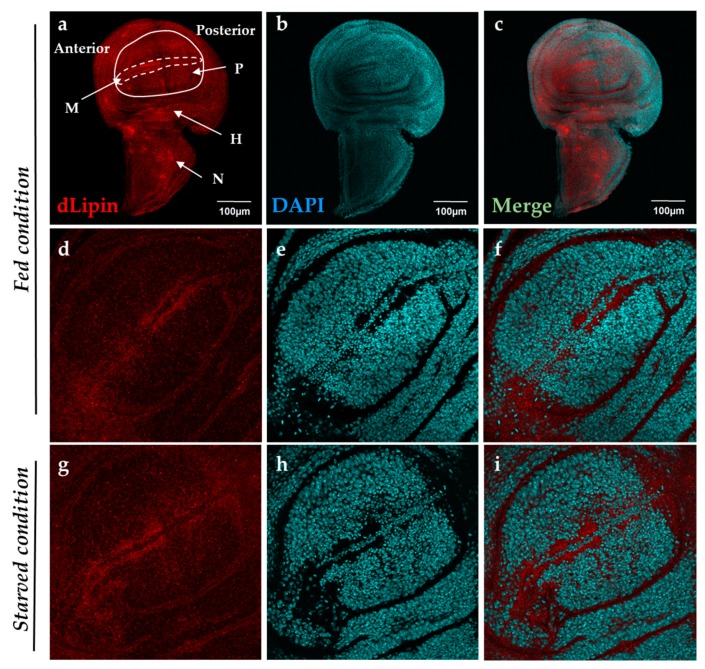
Localization of *Drosophila* lipin (dLipin) protein on the wing imaginal disc of the *yw* strain. Wing imaginal discs of 3rd-instar larvae were stained with 4′,6-diamidino-2-phenylindole (DAPI) (**b**,**e**,**h**) to visualize DNA, and rabbit anti-dLipin antibody (**a**,**d**,**g**) followed by anti-rabbit IgG Alexa Fluor^TM^ 594 antibody. Merged images of DAPI and antibody staining (**c**,**f**,**i**). Fed condition (**a**–**f**), starved condition (**g**–**i**). The images are representative among images of 10–20 wing imaginal discs. dLipin protein was expressed in whole wing imaginal disc, with particularly high expression in the anterior part of the margin (M), and notum (N), albeit slightly lower expression in the wing pouch (P) and hinge (H) (**a**–**c**). The dotted circle indicates the margin of the wing imaginal disc. The wing pouch demarcated with the white line is shown in (**d**,**g**). dLipin was not detected in the nuclei of wing imaginal discs of 3rd-instar larvae either in the fed or starved state (**d**–**i**). Scale bar, 50 µm.

It has been reported that dLipin resides on the wing imaginal disc of *D. melanogaster* [23]. To determine a specific dLipin location on the wing imaginal disc, we stained the wing imaginal discs from 3rd-instar larvae of the wild-type yellow-white (*yw*) strain with an anti-dLipin antibody. We found that dLipin signals are detected throughout the wing imaginal discs with relatively stronger signals in the anterior part of the margin and notum, and slightly lesser signals in the wing pouch and hinge (Figure 1a). In subsequent studies, we analyzed dLipin in the margin of the wing disc, which later becomes the wing margin of the adult wing blade. Lehmann’s group has reported that dLipin is translocated from the cytoplasm to nucleus in fat tissue under starvation conditions [23,24]. We also confirmed that dLipin is detected in both the cytoplasm and the nuclei of fat body cells in starved conditions by immunostaining (Figure S1). In contrast, immunostaining of wing imaginal discs from 3rd-instar larvae, in starved or fed condition, showed that unlike in fat tissue, dLipin did not appear to translocate into the nucleus of wing imaginal disc cells (Figure 1d–i).

### 2.2. Knockdown of dLipin Disrupts Normal Wing Pattern Formation

A previous study showed that lack of dLipin resulted in a lethal phenotype at late larval and pupal stages of *Drosophila* [23]. To determine whether dLipin is required for the development of specific tissues, two RNAi fly lines, UAS-*dLipin*-IR_277-380_ and *dLipin*-IR_265-272_, were crossed with various GAL4 driver lines that express GAL4 in selective tissues. Target sequences for these two RNAi sequences were designed to have no off-target effects (Vienna Drosophila Resource Center and online dsCheck software http://dscheck.rnai.jp). First, we overexpressed GFP using *Sd*-GAL4 drivers to confirm the region in the wing imaginal disc where GAL4 is expressed. Similar to previous reports [37,38,39], stronger GFP signals were detected in the margin area and wing pouch of 3rd-instar larvae driven by *Sd*-GAL4, suggesting effective knockdown of *dLipin* in this region of the knockdown fly (Figure S2). Furthermore, we checked the expression levels of dLipin mRNA and protein in the wing disc of 3rd-instar larvae by qRT-PCR and immunostaining, respectively. As shown in Figure S3, dLipin mRNA and protein levels were significantly reduced in the wing discs of knockdown fly lines, suggesting efficient knockdown of *dLipin* in wing imaginal discs of both knockdown flies.

**Figure 2:**
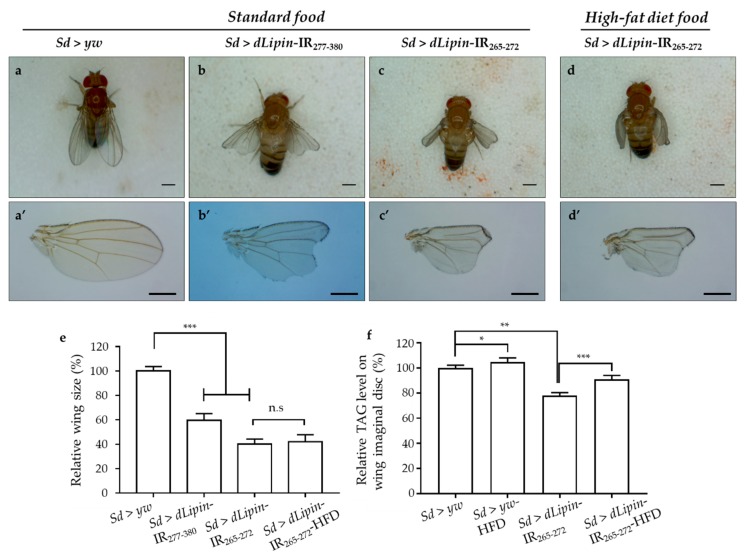
*dLipin*-kd selectively causes atrophied wing development in the wing imaginal disc. Micrographs of adult flies (**a**–**d**) and wing blades (**a’**–**d’**) are shown. The control flies were obtained by crossing the *Sd*-GAL4 drivers with the *yw* strain (**a**,**a’**). Wings of *dLipin*-kd flies (*Sd* > *dLipin*-IR_265-272_ and *Sd* > *dLipin*-IR_277-380_) showed wing notching and curl (**b**,**b’**,**c**,**c’**). *dLipin*-kd phenotypes were not recused by the administration of a high-fat diet (**d**,**d’**). The size of the wing blade was analyzed using ImageJ software. The relative size of the *dLipin*-kd fly wing to that of control fly are shown (*n* = 50 for each genotype) (**e**). The relative triacylglycerol (TAG) level of the *dLipin*-kd wing imaginal disc to that of the control was analyzed using 100 imaginal discs (*n* = 4 for each genotype) (**f**). Data are expressed as the means ± S.D. The statistical significance of the difference between control and *dLipin*-kd flies was evaluated using *t*-test and one-way ANOVA. Scale bar, 0.5 mm; *, *p* = 0.03, **, *p* < 0.02, ***, *p* < 0.01; n.s, no significant; IR, inverted repeat, HFD, high-fat diet. Genotypes: *Sd*-GAL4/y; +; + (**a**,**a’**), *Sd*-GAL4/y; UAS-*dLipin*-IR_277-380_/+; + (**b**,**b’**), *Sd*-GAL4/y; UAS-*dLipin*-IR_265-272_/+; + (**c**,**c’**,**d**,**d’**).

Having confirmed the efficient knockdown of dLipin in the margin area and wing pouch of 3rd-instar larvae driven by *Sd*-GAL4, we next observed the phenotype of wings of *dLipin*-knockdown flies. The phenotypic observation demonstrated that the two different lines with *dLipin*-knockdown (*dLipin*-kd; *Sd* > *dLipin*-IR_265-272_ and *Sd* > *dLipin*-IR_277-380_) in the wing disc mediated by *sd*-GAL4 led to atrophied wing formation, notched and down-curled wings, along with reduction in size (Figure 2b,b’,c,c’). Statistical analysis showed that the reduction of wing size by *dLipin*-kd was significant as compared to that of the control fly (Figure 2e). In contrast, the flies with *dLipin*-knockdown in the nervous system using the *elave*-GAL4 driver, or in hemocytes using *HmlΔ*-GAL4 or *He*-GAL4, showed no detectable phenotype, suggesting that *dLipin* has no or minimal role in the development of these tissues. However, the possibility of low-level expression of GAL4 protein leading to the insufficient *dLipin*-kd in these tissues could not be excluded. Thus, we confirmed that the *dLipin*-kd phenotypes were caused by the deficient expression of dLipin selectively in the margin area and wing pouch of the wing disc.

To confirm whether *dLipin*-kd phenotypes, notching, and curly wing blades were related to the deficient TAG level, we measured the TAG contents of whole-wing imaginal discs of control and knockdown flies. It was found that the TAG level was significantly reduced in *dLipin*-kd (Figure 2f), compared to that of the control. These data suggested that the atrophied wing blade formation might be related to the deficient TAG level in the wing imaginal disc. Then, to confirm this point, the *dLipin*-kd (*Sd* > *dLipin*-IR_265-272_) eggs were hatched on high-fat diet food, the hatched larvae were cultured on the same food, and then, the TAG levels of the whole-wing imaginal disc of 3rd-instar larvae and adult wing blades were analyzed. Notably, the *dLipin*-kd phenotypes were not rescued by high-fat diet food (Figure 2d,d’,e), even though TAG contents of wing discs of *Sd* > *dLipin*-IR_265-272_ flies were increased by high-fat diet. This demonstrated that the abnormal wing formation in *dLipin*-knockdown flies might not be caused by deficient TAG in the wing imaginal disc.

### 2.3. Knockdown of dLipin Inhibits Cells from Entering M Phase

**Figure 3:**
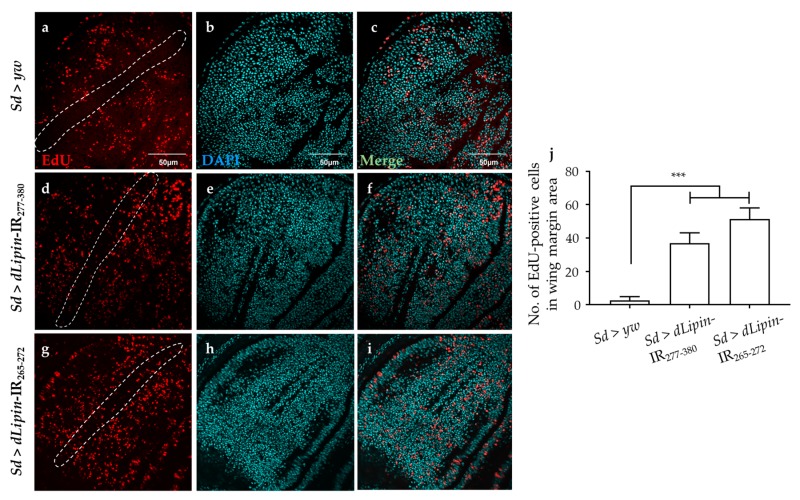
Knockdown of *dLipin* induces accumulation of cells in S phase. Wing imaginal discs from 3rd-instar larvae of control (*Sd* > *yw*) and *dLipin*-kd flies (*Sd* > *dLipin*-IR_265-272_ and *Sd* > *dLipin*-IR_277-380_) were stained with DAPI to visualize the DNA (**b**,**e**,**h**) and click-iT 5-Ethynyl-2′-deoxyuridine (EdU) Alexa Fluor^TM^ 594 (**a**,**d**,**g**). Merged images of DAPI and EdU staining are shown (**c**,**f**,**i**). The number of EdU-positive cells in S phase in the wing margin area of 3rd-instar larvae, circled with a dotted line, were analyzed using MetaMorph software (*n* = 10 for each genotype) (**j**). Dotted line indicates the margin of the wing imaginal disc. Data are expressed as the means ± S.D. The statistical significance of the difference between control and *dLipin*-kd flies was evaluated using one-way ANOVA. Scale bar, 50 μm. ***, *p* < 0.01. IR, inverted repeat. Genotypes: *Sd*-GAL4/+; +; + (**a**–**c**), *Sd*-GAL4/+; UAS-*dLipin*-IR_277-380_/+; + (**d**–**f**), *Sd*-GAL4/+; UAS-*dLipin*-IR_265-272_/+; + (**g**–**i**).

To reveal the mechanism underlying the aberrant wing formation in *dLipin*-kd flies, we analyzed the effect of *dLipin* knockdown on the cell cycle progression in wing imaginal discs. We first determined the number of cells in S phase by 5-Ethynyl-2′-deoxyuridine (EdU) pulse labeling [40], and found a significant increase in the EdU-positive cell in the wing margin of *dLipin*-kd wing imaginal discs (Figure 3d,g,j) as compared to that in the control flies (Figure 3a,j).

**Figure 4:**
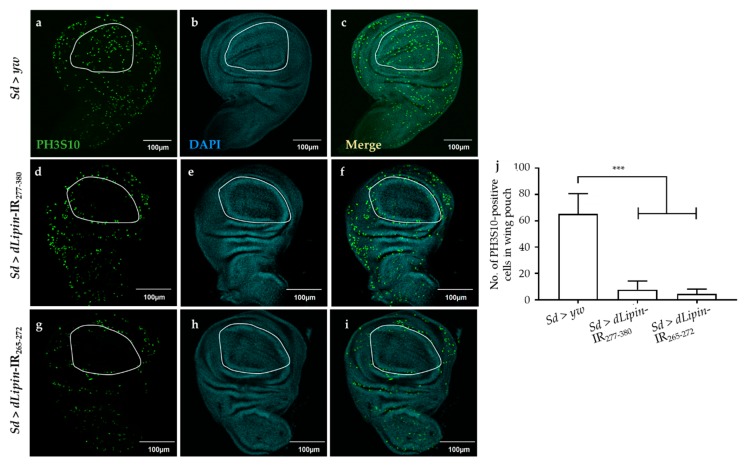
Knockdown of *dLipin* leads to a reduced number of mitotic cells in wing pouch. The wing imaginal discs from 3rd-instar larvae of control (*Sd* > *yw*) and *dLipin*-kd flies (*Sd* > *dLipin*-IR_265-272_, *Sd* > *dLipin*-IR_277-380_) were stained with DAPI to visualize the DNA (**b**,**e**,**h**) and anti-PH3S10 antibody followed by anti-rabbit IgG Alexa Fluor^TM^ 488 antibody (**a**,**d**,**g**). Merged images of DAPI and PH3S10 antibody staining are shown (**c**,**f**,**i**). PH3S10-positive cells (mitotic cells) in the wing pouch of 3rd-instar larvae were counted using MetaMorph software (*n* = 10 for each genotype) (**j**). Dotted line indicates the wing pouch of the wing imaginal disc. Data are expressed as the means ± S.D. The statistical significance of the difference between control and *dLipin*-kd flies was evaluated using one-way ANOVA. ***, *p* < 0.01; Scale bar, 100 μm; IR, inverted repeat. Genotypes: *Sd*-GAL4/+; +; + (**a**–**c**), *Sd*-GAL4/+; UAS-*dLipin*-IR_277-380_/+; + (**d**–**f**), *Sd*-GAL4/+; UAS-*dLipin*-IR_265-272_/+; + (**g**–**i**).

We next analyzed the number of mitotic cells by immunostaining the wing imaginal discs with an anti-PH3S10 antibody, which is a hallmark of initiation of mitosis [41,42]. Compared to the control (Figure 4a), the wing imaginal disc of *dLipin*-kd flies showed a significantly reduced number of PH3S10-positive cells in the wing margin and wing pouch (Figure 4d,g,j). Taken together with the increased cells in S phase, the reduced number of mitotic cells suggested that dysfunction of *dLipin* suppresses cell cycle transition from S to M phase in the wing imaginal disc, possibly owing to G2/M checkpoint activation.

### 2.4. Dysfunction of dLipin Leads to Down-Regulated Expression of Cyclin B (CycB)

**Figure 5:**
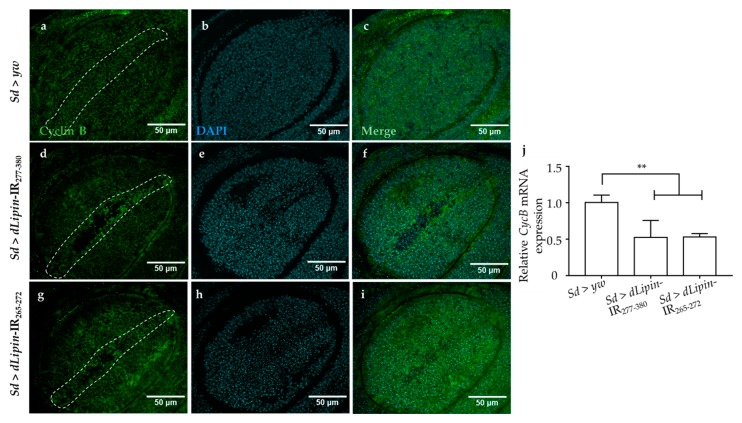
Induction of *dLipin* RNAi leads to reduced expression of the cyclin B in the wing margin area of wing imaginal discs. The wing imaginal discs were stained with DAPI to visualize the DNA (**b**,**e**,**h**) and mouse anti-cyclin B antibody followed by anti-mouse IgG Alexa Fluor^TM^ 488 antibody (**a**,**d**,**g**). Merged images of DAPI and anti-cyclin B antibody (**c**,**f**,**i**). *CycB* mRNA levels in wing imaginal discs of 3rd-instar larvae of control and *dLipin*-kd flies were analyzed by RT-qPCR (*n* = 5 for each genotype). The relative *dLipin* mRNA level of *dLipin*-kd flies to that of control flies is shown (**j**). Data are expressed as the means ± S.D. The statistical significance of the difference between control and *dLipin*-kd flies was evaluated using one-way ANOVA. **, *p* < 0.02. The dotted lines indicate the margins of wing imaginal discs. Scale bar, 50 μm; IR, inverted repeat. Genotypes: *Sd*-GAL4/+; +; + (**a**–**c**), *Sd*-GAL4/+; UAS-*dLipin*-IR_277-380_/+; + (**d**–**f**), *Sd*-GAL4/+; UAS-*dLipin*-IR_265-272_/+; + (**g**–**i**).

The CycB-CDK1 complex is necessary for the transition from G2 to M phase, and cyclin B expression peaks during late G2 and early mitosis [43,44,45]. We speculated that the G2/M checkpoint in *dLipin*-kd flies might be activated by dysregulated expression of cyclin B. To test this hypothesis, we checked the mRNA and protein levels of cyclin B in the wing imaginal discs of 3rd-instar larvae of the *dLipin*-kd strain by qRT-PCR and immunostaining with an anti-cyclin B antibody, respectively. As hypothesized, *CycB* mRNA was significantly reduced in both the knockdown flies (Figure 5j). Additionally, cyclin B protein level was decreased in the margin area of wing imaginal discs of *dLipin*-kd flies (Figure 5d,g), compared to that in the control flies (Figure 5a). These results suggested that the dysfunction of dLipin may lead to reduced expression of cyclin B and, subsequently, to activation of the G2/M checkpoint.

### 2.5. Knockdown of dLipin Causes DNA Damage-Induced Apoptotic Cell Death

**Figure 6:**
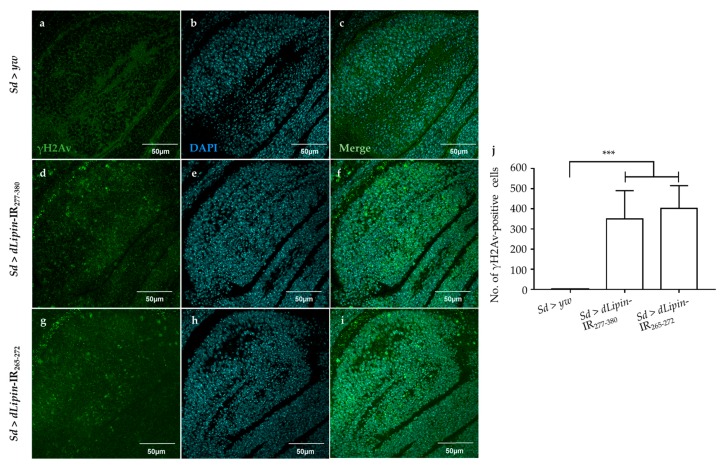
Knockdown of *dLipin* causes DNA damage in the wing pouch. Control and *dLipin*-kd wing imaginal discs were stained with DAPI to visualize the DNA (**b**,**e**,**h**) and mouse anti-γH2Av antibody followed by anti-mouse IgG Alexa Fluor^TM^ 488 antibody (**a**,**d**,**g**). Merged images of DAPI and anti-γH2Av antibody staining (**c**,**f**,**i**). The number of γH2Av-positive cells in the wing pouch of imaginal discs of 3rd-instar larvae from control and *dLipin*-kd flies was analyzed using MetaMorph software (*n* = 14 for each genotype) (**j**). Data are expressed as the means ± S.D. The statistical significance of the difference between control and *dLipin*-kd flies was evaluated using one-way ANOVA. ***, *p* < 0.01. Scale bar, 50 μm; IR, inverted repeat. Genotypes: *Sd*-GAL4/+; +; + (**a**–**c**), *Sd*-GAL4/+; UAS-*dLipin*-IR_277-380_/+; + (**d**–**f**), *Sd*-GAL4/+; UAS-*dLipin*-IR_265-272_/+; + (**g**–**i**).

Activation of the G2/M checkpoint is known to prevent the cells from initiating mitosis when DNA damage occurs during G2, or when cells progress into G2 with some unrepaired damage inflicted during the previous S phase [46]. Upon DNA damage, both CycB transcription and protein level are down-regulated [47,48]. To clarify whether the activation of the G2/M checkpoint in *dLipin*-kd flies was induced by DNA damage, we examined the expression of histone variant H2Av phosphorylated at Ser137 (γH2Av), the homolog of mammalian histone variant H2AX [49,50], which is a marker for an early signal of DNA damage induced by replication stress [51,52]. As shown in Figure 6d,g,j, *dLipin*-kd flies showed a markedly increased number of γH2Av-positive cells, indicating that the G2/M checkpoint was activated by DNA damage.

**Figure 7:**
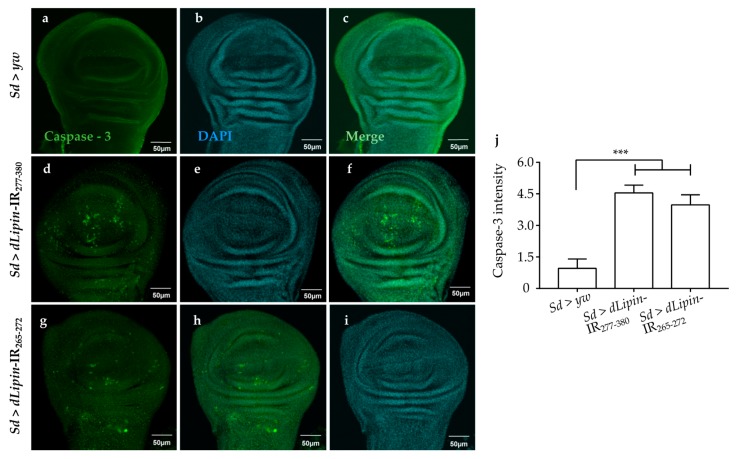
Knockdown of *dLipin* causes caspase-dependent cell death in wing imaginal discs of *Drosophila*. Wing imaginal discs from 3rd-instar larvae of control and *dLipin*-kd flies were stained with DAPI to visualize the DNA (**b**,**e**,**h**), and with rabbit anti-cleaved caspase-3 antibody followed by anti-rabbit IgG Alexa Fluor^TM^ 488 antibody (**a**,**d**,**g**). Merged images of DAPI and anti-cleaved caspase-3 antibody are shown (**c**,**f**,**i**). The fluorescence intensity in the wing pouch stained with anti-cleaved caspase-3 antibody was analyzed using MetaMorph software (*n* = 14 for each genotype) (**j**). Data are expressed as the means ± S.D. The statistical significance of the difference between control and *dLipin*-kd flies was evaluated using one-way ANOVA. ***, *p* < 0.01; Scale bar, 50 μm. IR, inverted repeat. Genotypes: *Sd*-GAL4/+; +; + (**a**–**c**), *Sd*-GAL4/+; UAS-*dLipin*-IR_277-380_/+; + (**d**–**f**), *Sd*-GAL4/+; UAS-*dLipin*-IR_254-476_/+; + (**g**–**i**).

Next, we examined whether knockdown of *dLipin* enhanced apoptotic cell death following DNA damage. Upon immunostaining, *dLipin*-kd flies showed significantly increased cleaved caspase-3 signal in the wing pouch of wing imaginal discs (Figure 7d,g,j) in comparison to that in the controls (Figure 7a,j).

**Figure 8:**
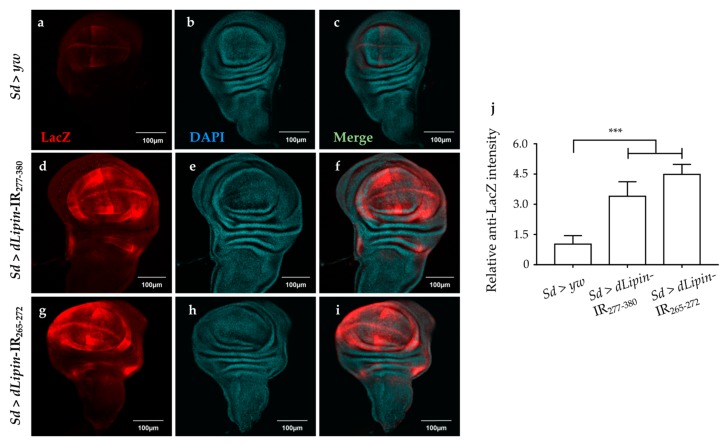
Knockdown of *dLipin* activates the pro-apoptotic gene *reaper*. Wing imaginal discs from the 3rd-instar larvae of control and *dLipin*-kd flies that carry *rpr*-lacZ were stained with an anti-lacZ antibody (**a**,**d**,**g**) and with DAPI (**b**,**e**,**h**). Both images were merged (**c**,**f**,**i**). The fluorescence intensities in the wing pouch stained with anti-lacZ were analyzed using MetaMorph software (*n* = 14 for each genotype) (**j**). Data are expressed as the means ± S.D. The statistical significance of the difference between control and *dLipin*-kd flies was evaluated using one-way ANOVA. ***, *p* < 0.01; Scale bar, 100 μm; IR, inverted repeat. Genotypes: *Sd*-GAL4/+; +; *rpr*-lacZ/+ (**a**–**c**), *Sd*-GAL4/+; UAS-*dLipin*-IR_277-380_/+; *rpr*-lacZ/+ (**d**–**f**), *Sd*-GAL4/+; UAS-*dLipin*-IR_254-476_/+; *rpr*-lacZ/+ (**g**–**i**).

We analyzed the expression of the pro-apoptotic gene *reaper* (*rpr*) by using *dLipin*-kd flies that carry *rpr*-lacZ as a reporter. The results demonstrated that *dLipin* knockdown in the margin area and wing pouch of wing imaginal disc, driven by *Sd*-GAL4, resulted in significantly up-regulated transcription of *rpr* (Figure 8d,g). In addition, we established *dLipin*-kd flies, in which death-associated inhibitor of apoptosis 1 (DIAP1) was overexpressed by the *Sd*-GAL4 driver, to examine whether the phenotype of *dLipin*-kd could be rescued. The *dLipin* knockdown phenotypes were partially rescued by *diap1* overexpression (Figure S4). The cleaved caspase-3 and anti-lacZ signals detected outside of the expression domain of *Sd*-GAL4 in Figure 7d,g and Figure 8d,g could be explained by the non-cell autonomy. These results suggested that dysfunction of *dLipin* causes DNA damage-induced apoptotic cell death in the wing imaginal disc of *Drosophila*.

## 3. Discussion

Lipin reportedly has three main functions: As an enzyme catalyzing the production of DAG from PA, maintaining a balance between PA and DAG, and as an inducible transcriptional coactivator in conjunction with PPARγ to regulate several lipid metabolism-related genes [9,12]. Previous studies demonstrated that dLipin can be detected in wing imaginal discs [23], and is necessary for wing vein formation via BMP signaling [53]. However, there have been no reports regarding the functions of dLipin in wing blade formation. In the present study, we found that dLipin could be detected in the wing imaginal disc with a higher level in the margin of the wing pouch and the notum region, which become the wing margin and thorax of adult flies, compared to that in other areas. Knockdown of *dLipin* led to wing notching, down-curled wing, and significantly smaller wing size (Figure 2), suggesting the important role of dLipin in the normal formation of the wing blade. Furthermore, we showed that the abnormal formation of the wing in *dLipin*-kd flies is caused by the inhibition of the transition from S phase to M phase during the cell cycle.

A previous study demonstrated that mutation of dLipin induces reduced levels of TAG, which plays a central role in cellular lipid storage in invertebrates, in whole-larvae of *Drosophila* [23]. We hypothesized that knockdown of *dLipin* in the wing imaginal disc may affect the production of DAG from PA. Deficiency of DAG, which is a precursor to TAG, leads to deficient TAG content, thereby affecting the development of adult wing blades. However, our results of high-fat diet administration demonstrated that the curly and notched wings are not likely to be caused by the deficiency of TAG in wing imaginal discs of *dLipin*-kd flies (Figure 2). Dwyer et al. reported that PA and DAG are required for the biosynthesis of phospholipids [4], which are mainly found in cell membranes and play pivotal roles in cell physiology [2,54]. Therefore, the deficiency of DAG may affect the cell cycle process, thereby disrupting wing formation. Moreover, lipin is a key regulator of nuclear membrane growth during the cell cycle in yeast [11]. Jackowski reported that phospholipids accumulated in the S phase [55], suggesting that the decrease in DAG by dLipin dysfunction may affect the cell cycle in S phase. Consistent with this, we found that *dLipin*-kd in the wing pouch of 3rd-instar larvae induced the accumulation of cells in S phase (Figure 3).

Laskye et al. showed that the entire DNA content in the nucleus must be completely and precisely replicated during the S phase of the cell cycle [56]. Earlier studies reported that phospholipids (i.e., cardiolipin, PA, phosphatidylglycerol, and phosphatidylinositol) inhibited DNA replication in mitochondria and the nucleus through interaction with DNA polymerases [7,57]. Notably, lipin can regulate the syntheses of phosphatidylcholine and other phospholipids by repressing key genes of the biosynthesis pathway in yeast [11]. It is; thus, possible that the increased PA in wing imaginal discs of *dLipin*-kd flies may inhibit the interaction between DNA polymerases and phospholipids, thereby causing DNA replication stress by generating incompletely replicated DNA.

The accumulation of cells in S phase in the wing margin of *dLipin*-kd wing imaginal discs might be associated with DNA damage induced by replication stress [58]. Moreover, our results showed that the wing margin and wing pouch of *dLipin*-kd wing imaginal discs exhibited increased expression of the γH2Av protein, a marker for DNA damage induced by replication stress (Figure 6). In addition, Ugrankar et al. reported that loss of *dLipin* in the fat body caused nuclei fragmentation [23]. Together, this evidence indicated that the accumulation of cells in S phase in the wing margin of 3rd-instar larvae of *dLipin*-kd strain was due to DNA damage in the cell cycle process.

The accuracy of DNA replication and division is facilitated by cell cycle DNA damage checkpoints [59,60], which are located at specific positions in the cell cycle to detect damage and allow sufficient time for DNA repair [34,35,36]. Our observations showed that the number of mitotic cells in M phase was significantly reduced in the wing pouch of *dLipin*-kd flies (Figure 4). In addition, both transcription and protein levels of cyclin B were reduced in the wing imaginal disc of *dLipin*-kd flies, indicating activation of the G2/M checkpoint in these flies (Figure 5). We; therefore, concluded that the G2/M checkpoint was activated in response to DNA damage in these flies. It was also found that the cyclin B intensities of several cells located near the wing margin region of *dLipin*-kd flies were higher than those of the control. It is possible that elevation of the cyclin B level of neighboring cells may compensate for lack of cyclin B of cells in the wing margin of knockdown flies, although currently the mechanism underlying this response remains to be elucidated. There are four distinct pathways related to DNA damage response: transcriptional induction, cell cycle arrest (also known as DNA damage checkpoint), DNA repair, and apoptosis. These pathways work independently under certain conditions, but frequently interact to repair the damaged DNA or activate apoptosis [61,62,63,64]. We found increased signals of anti-cleaved caspase-3 antibody (Figure 7) and elevated expression of *reaper* gene (Figure 8) in the wing pouch of *dLipin*-kd flies. Taken together, these results implicated that dysfunction of *dLipin* might lead to apoptotic cell death induced by DNA damage in the wing imaginal disc of *Drosophila*.

In a state of starvation, dLipin in the fat tissue is translocated from the cytoplasm to the nucleus [23,24]. Lack of TORC1 leads to translocation of lipin-1 into the nucleus in mammalian cells [25]. In mouse, lipin-1 works as a transcriptional co-regulator and directly controls the gene encoding nuclear receptor PPARα, whereas lipin-1 overexpression causes the activation of genes related to fatty acid transport, β-oxidation, the TCA cycle, and oxidative phosphorylation, including many target PPARα genes [11,65]. This suggests that lipin-1 directly regulates genes to overcome energy deficiency during starvation. However, we could not detect dLipin signals in the nucleus of wing imaginal disc cells in starvation conditions. In addition, it is as yet unclear what genes are regulated by nuclear dLipin in *Drosophila* and the mechanism thereof. Further studies are necessary to answer these questions. The translocation of mammalian lipin-1 into the nucleus may affect gene expression through an unknown PAP-dependent mechanism that regulates nuclear levels of the transcription factor SREBP-1, which regulates the expression of genes related to lipid homeostasis [21]. Sethi et al. reported that SREBP-1 serves as a bridge between lipogenesis and cell cycle progression of clear cell renal carcinoma [66]. Thus, we could not exclude the possibility that *dLipin*-kd may indirectly inhibit SREBP-1 expression in the wing imaginal disc, thereby affecting cell cycle proliferation.

In conclusion, knockdown of *dLipin* in the wing imaginal disc of *Drosophila* causes DNA damage. The DNA damage activates the G2/M DNA damage checkpoint by regulating cyclin B expression, inhibiting the transition from S phase to M phase. Furthermore, *dLipin* dysfunction may lead to apoptosis of cells in the wing imaginal disc of *D. melanogaster*, leading to the formation of wing notching and a significantly smaller wing. This is the first report regarding the function of dLipin in wing development.

## 4. Materials and Methods

### 4.1. Fly Stocks

Fly stocks were maintained at 25 °C on standard food. Transgenic flies carrying UAS-*dLipin*-IR_265-272_ and UAS-*dLipin*-IR_277-380_ were obtained from the Bloomington Drosophila Stock Center (BDSC) and Vienna Drosophila Resource Center (VDRC), respectively. These flies carry an inverted repeat (IR) of the *lipin* gene (targeting regions from amino acid 265 to 272 and from 277 to 380, respectively) downstream of the UAS sequence, on the second chromosome. Target sequences for these two RNAi sequences were designed to have no off-target effects (VDRC and online dsCheck software http://dscheck.rnai.jp). All other flies used in this study were obtained from BDSC. The *yw* flies were used as the wild-type strain.

### 4.2. Staining

The wandering 3rd-instar larvae were dissected in PBS to collect wing imaginal discs. Approximately 20 to 30 wing imaginal discs were fixed in 4% formaldehyde in PBS and reacted with antibodies as described previously [67]. Anti-dLipin antibody [20], provided kindly by Prof. Dr. Michael Lehmann (University of Arkansas, Arkansas, USA), was used at a 1:3000 dilution, and then anti-rabbit IgG Alexa Fluor^TM^ 594 (Molecular Probes, Invitrogen, Carlsbad, CA, USA) was used at a dilution of 1:800. The wing imaginal discs were respectively treated with anti-histone H3 (phospho S10), anti-cleaved caspase-3 IgG, anti-LacZ (Cell Signaling Technology (CST), Tokyo, Japan), anti-cyclin B, and anti-γH2Av antibodies (Santa Cruz Biotechnology, Dallas, TX, USA) at a 1:600 dilution, followed by incubation with Alexa Fluor^TM^ 488-conjugated anti-mouse IgG at a dilution of 1:800. For nuclei staining, 4′,6-diamidino-2-phenylindole (DAPI; Molecular Probes, Eugene, OR, USA) was used.

Male and female transgenic fly were mated and kept for 1 day at 25 °C, then transferred to a new standard food tube for 1 h to deposit eggs to obtain a synchronized larval age. At the desired period of larval growth, wing imaginal discs were collected for assays. 5-Ethynyl-2′-deoxyuridine (EdU) labeling was performed according to the manufacturer’s instructions (Molecular Probes).

After reacting with antibody, DAPI, or EdU, wing imaginal discs were mounted on a glass slide in Vectashield mounting medium (Vector Laboratories, Tokyo, Japan), and then inspected using a fluorescence FV10i microscope (Olympus, Tokyo, Japan). The fluorescence intensity in the wing pouch was analyzed using MetaMorph software (version 7.7.7.0; Molecular Devices, Sunnyvale, CA, USA), and the intensity in the wing pouch was subtracted from that of the area outside.

### 4.3. Starvation Assay

Pre-wandering 3rd-instar larvae of the *yw* strain were transferred to either standard food (fed larvae) or cotton plugs soaked in PBS (starved larvae). After 4 h, the wing imaginal discs were dissected out and reacted with anti-dLipin antibody as described above [24].

### 4.4. High-Fat Diet and Triglyceride Assays

The standard food supplement contained 0.8% agar (*w*/*v*), 9% cornmeal (*w*/*v*), 4% dry yeast (*w*/*v*), 0.05% (*w*/*v*) ethyl *p*-hydroxybenzoate, and 0.5% propionic acid (*v*/*v*). For preparation of the high-fat diet, we added 20% (*w*/*v*) of food-grade coconut oil [68]. Five male and female transgenic flies were mated and allowed to lay eggs on the high-fat diet food for 2 days at 25 °C. The hatched larvae were grown on the same diet.

TAG contents were measured using the infinity triglycerides assay kit (Thermo Fisher Scientific, Waltham, MA, USA). Exactly 100 wing imaginal discs of wandering 3rd-instar larvae of each sample were dissected out and placed into tubes. The tubes were either placed on ice immediately for the assay, or stored at −80 °C for later assessment. Wing imaginal discs were homogenized in 100 µL of PBS containing 0.3 % Triton X-100. Homogenates were heated to 70 °C for 5 min, and then centrifuged at 16,150× *g* for 1 min at room temperature. The supernatant was transferred and centrifuged again at 30,050× *g* and 4 °C for 5 min. The final supernatant was assayed for TAG content. Briefly, 5 µL of supernatant was added to 200 µL of triglyceride reagent in the assay kit, and the mixture was incubated at 37 °C for 5 min. The optical density at 520 nm (OD_520_) was measured, and TAG values were calculated according to the manufacturer’s instructions by using glycerol standards for calibration.

### 4.5. Quantitative RT-PCR

Total RNA was extracted from 40 wing imaginal discs using standard Qiazol reagent (Qiagen, Hilden, Germany) followed by purification with the Qiagen RNeasy kit. cDNA was synthesized using the SimpliAmp™ Thermal Cycler (Life Technologies, Singapore, Singapore) according to the instruction manual. Quantitative polymerase chain reaction (PCR) was performed using the FastStart Essential DNA Green Master Mix (Roche, Mannheim, Germany) and a LightCycler 96 (Roche). *rp49* was used as an internal control. The sequences of gene-specific primers were as following: *dLipin*, forward: 5′-ATCCCACGTCCCTGATATCG-3′ and reverse: 5′-TTCATCTTGGTTGGTTAGCAGG-3′; for *CycB*, forward: 5′-GGATGCGGCACAGAAAGA-3′ and reverse: 5′-CTGTCCACCCGAGCTTTG-3′; for *rp49*, forward: 5′-ACCAGCTTCAAGATGACCATCC-3′ and reverse: 5′- CTTGTTCGATCCGTAACCGATG-3′.

### 4.6. Statistical Analysis

The experiments were repeated at least three times. The data are expressed as means ± S.D. The statistical significance of differences was evaluated using a *t*-test and one-way ANOVA. The *p*-values of <0.05 were considered significant.

## 5. Conclusions

Based on our results, we suggest that dLipin is necessary for the cell cycle progression subsequent to normal DNA replication during wing development of *D. melanogaster*. Further studies are required to understand the role of *dLipin* in the G2/M checkpoint and the expression of several genes implicated in DNA damage and repair. Moreover, a previous study demonstrated that the overexpression of *dLipin* in the Drosophila *dullard*, *ddd* hypomorphic mutant background rescues the atrophic wing vein phenotypes of the *ddd* mutant, indicating that the relationship between *dLipin* and *Dullard* is conserved in *Drosophila* [53]. Taken together, these results suggest that the balance of Lipin expression, which functions as an enzyme in the cytoplasm, is necessary for normal development of *D. melanogaster*.

## Supplementary Materials

Supplementary materials can be found at https://www.mdpi.com/1422-0067/20/13/3288/s1.
