# Supplementary material for: The Function of Lipin in the Wing Development of Drosophila melanogaster
Source: Int J Mol Sci. 2019 Jul 4;20(13):3288. doi: 10.3390/ijms20133288 (PMC6650997; doi:10.3390/ijms20133288)
Supplement: Supplementary file 1 [file ijms-20-03288-s001.pdf]

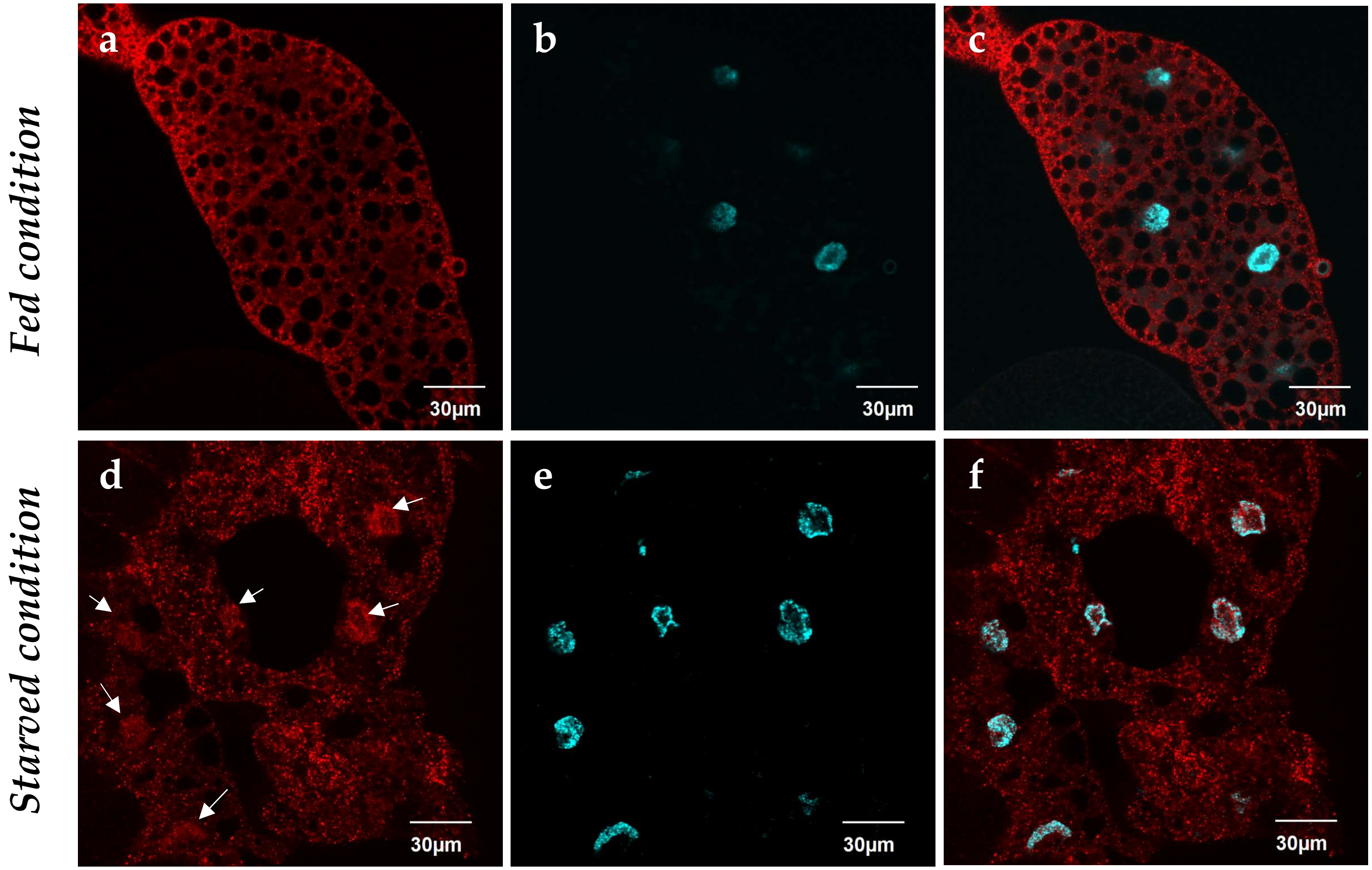

Figure S1. Localization of dLipin protein on the fat body of *D. melanogaster*. The fat bodies of the 3rd-instar larvae of the *yw* strain were stained with DAPI (**b, e**) to visualize DNA, and rabbit anti-dLipin antibody followed by anti-rabbit IgG Alexa Flour™ 594 antibody (**a, d**). Merged images of DAPI and antibody staining (**c, f**). Localization of dLipin protein was shown on fat body cells in fed (**a – c**) and starved (**d – f**) conditions, respectively. dLipin signals detected in the nuclei of fat body cells under starved condition are shown by arrows (**d**), while no dLipin signal was detected under fed condition (**a**).

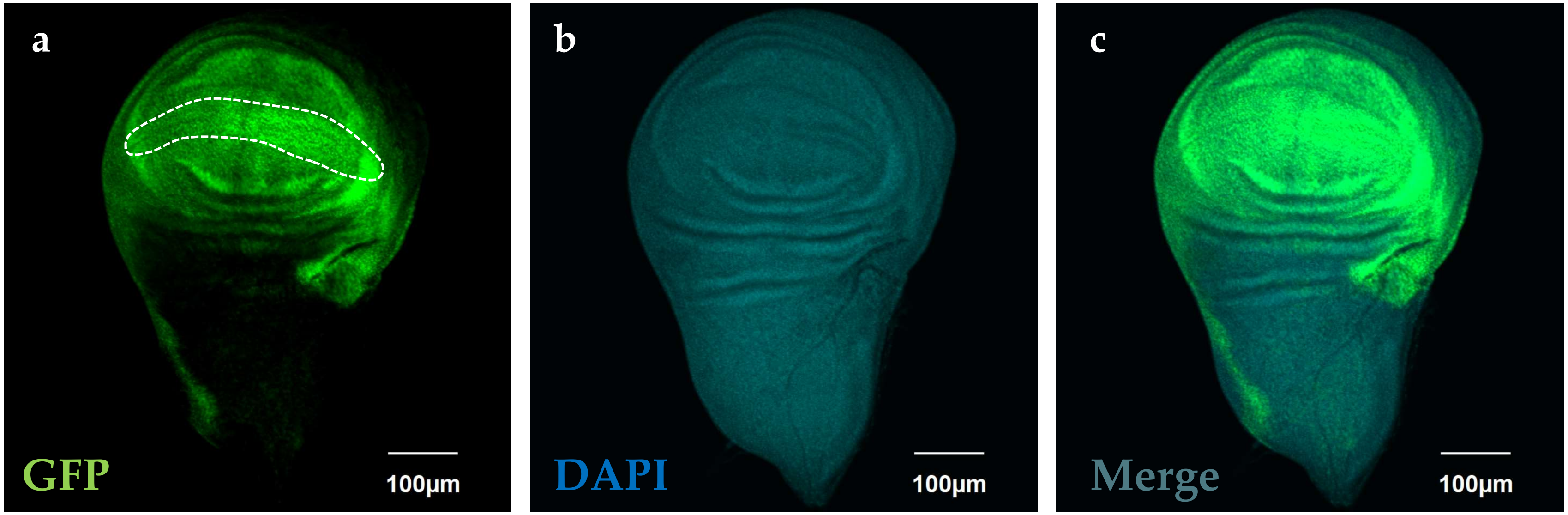

Figure S2. Expression regions of GAL4 in *Sd*-GAL4 driver line. To confirm expression region of GAL4 protein driven by Sd promoter, Sd-GAL4 driver line was crossed with flies possessing UAS-GFP gene. The expression of GFP in wing pouch of 3rd-instar larvae was visualized by fluorescence (**a**), and wing imaginal discs were stained with DAPI to detect DNA (**b**). Merge image of GFP fluorescence and DAPI staining is shown in (**c**) . Dotted circle indicates margin area of wing imaginal disc. Scale bar, 100 µm. Genotype: Sd-GAL4/+;+; UAS-GFP/+.

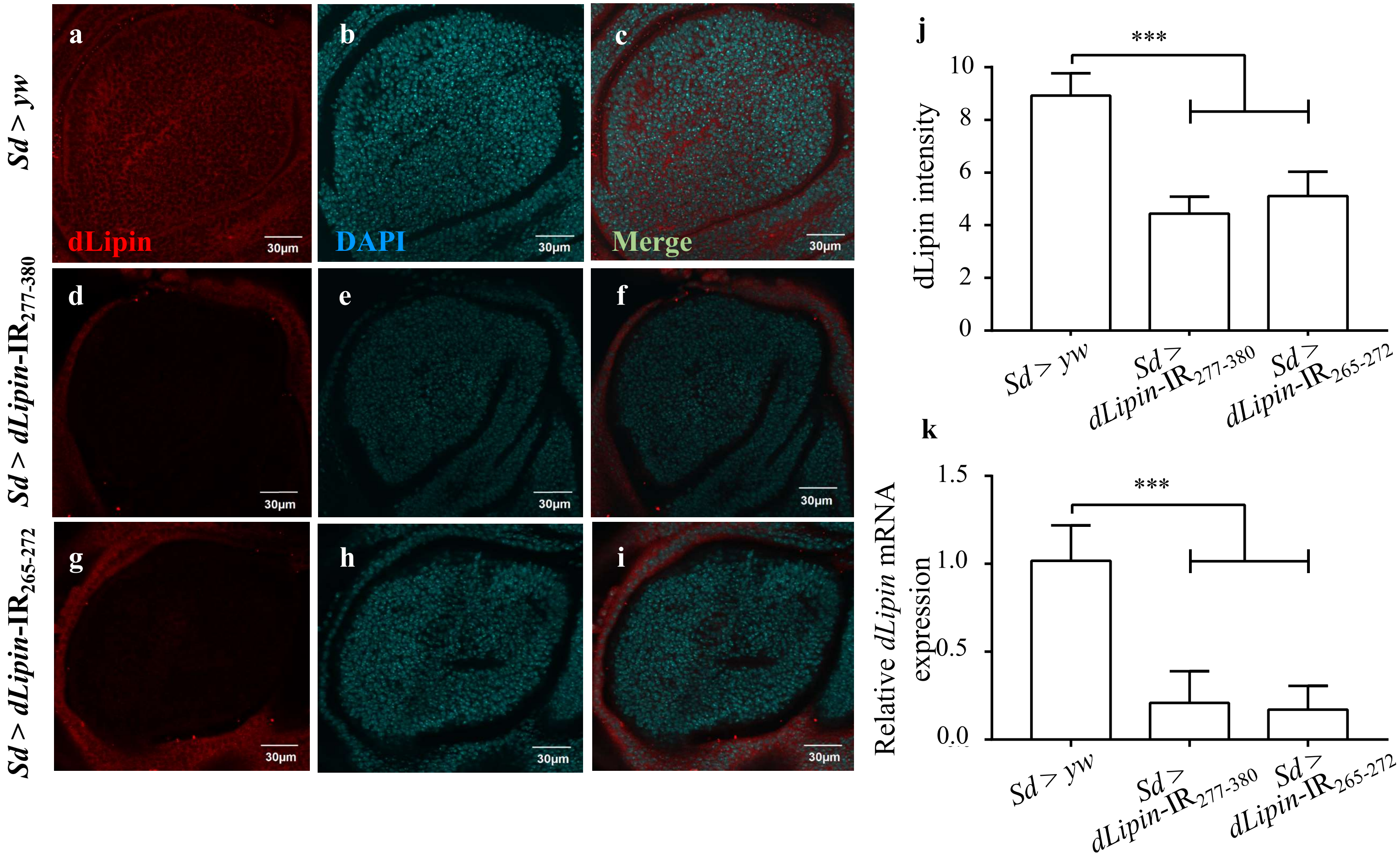

Figure S3. Induction of dLipin RNAi leads to reduced expression of dLipin. Wing imaginal discs of control and *dLipin*-kd flies were stained with DAPI to visualize DNA (**b, e, h**), and treated with rabbit anti-dLipin antibody followed by anti-rabbit IgG Alexa FlourTM 594 antibody (**a, d, g**). Merge images of both images of DAPI staining and immunostaining are shown (**c, f, i**). The fluorescence intensities in the wing pouch stained with anti-dLipin antibody were analyzed using MetaMorph software (n = 25 for each genotype) (**j**). dLipin mRNA levels in wing imaginal discs of 3rd-instar larvae of control and *dLipin*-kd flies were analyzed by RT-qPCR (n = 5 for each genotype), and relative dLipin mRNA level of *dLipin*-kd flies to that of control flies are shown (**k**). Data are expressed as the mean ± S.D. The statistical significance of the difference between control and *dLipin*-kd flies was evaluated using one-way ANOVA. ***, $p < 0.01$; Scale bar, 30 μm; IR, inverted repeat. Genotypes: *Sd*-GAL4/+; +; + (**a - c**), *Sd*-GAL4/+; UAS-*dLipin*-$IR_{277-380}$/+; + (**d - f**), *Sd*-GAL4/+; UAS-*dLipin*-$IR_{265-272}$/+; + (**g - i**).

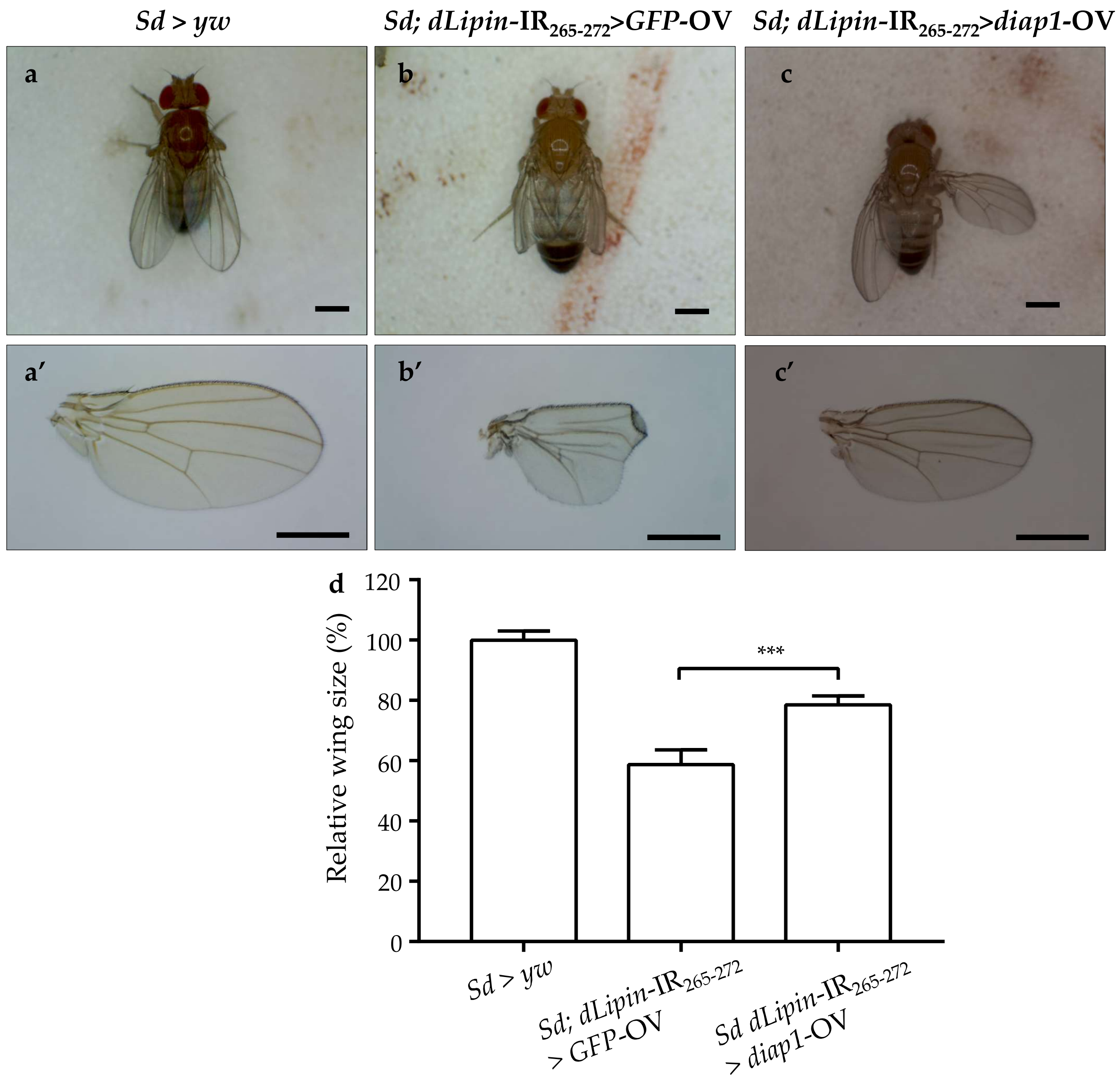

Figure S4. *dLipin*-kd phenotypes are suppressed by overexpression of an apoptosis inhibitor. Micrographs of adult flies (**a - c**) and wing blade (**a′ - c′**) are shown. *dLipin*-kd-induced curl, notched wings were partially suppressed by the overexpression of *diap1* (**c, c′, d**), but remained unaffected by *gfp* overexpression (**b, b′, d**). Data are expressed as the mean ± S.D. The statistical significance of the difference between *dLipin*-kd and control flies were evaluated using a t test for two samples assuming equal variances (n = 50 for each phenotype) (**d**). Scale bar, 0.5 mm; ***, $p \leq 0.01$, IR, inverted repeat; OV, overexpression. Genotypes: *Sd*-GAL4/y; +; + (**a, a′**), *Sd*-GAL4/y; UAS-*dLipin*-IR$_{265\text{-}272}$/+; UAS-*GFP*/+ (**b, b′**), *Sd*-GAL4/y; UAS-*dLipin*-IR$_{265\text{-}272}$/+; UAS-*diap1*/+ (**c, c′**).
